# The introduction of a safety checklist in two UK hospital emergency departments: A qualitative study of implementation and staff use

**DOI:** 10.1111/jocn.15184

**Published:** 2020-01-30

**Authors:** Tracey Stone, Jon Banks, Heather Brant, Joanna Kesten, Emma Redfern, Ann Remmers, Sabi Redwood

**Affiliations:** ^1^ The National Institute for Health Research Applied Research Collaboration West at University Hospitals Bristol NHS Foundation Trust Bristol UK; ^2^ Population Health Sciences Bristol Medical School University of Bristol Bristol UK; ^3^ Health Protection Research Unit in Evaluation of Interventions The National Institute for Health Research University of Bristol Bristol UK; ^4^ University Hospitals Bristol NHS Foundation Trust Bristol UK; ^5^ West of England Academic Health Science Network Bristol UK

**Keywords:** assessment, communication, emergency care, emergency department, implementation, patient safety, rounding

## Abstract

**Aims and objectives:**

To explore the extent to which a checklist designed to support patient safety in hospital Emergency Departments was recognised and used by staff.

**Background:**

Patient crowding in UK Emergency Departments makes it difficult for staff to monitor all patients for signs of clinical deterioration. An Emergency Department Safety Checklist was developed at a UK hospital to ensure patients are regularly monitored. It was subsequently implemented in six hospitals and recommended for use across the National Health Service in England.

**Methods:**

This was a qualitative study in two UK hospital Emergency Departments. Data collection consisted of sixty‐six hours of nonparticipant observation and interviews with twenty‐six staff. Observations were sampled across different days and times. Interviews sampled a range of staff. Data were analysed thematically. The study was undertaken in accordance with COREQ guidelines.

**Results:**

Staff described the Emergency Department Safety Checklist as a useful prompt and reminder for monitoring patients' vital signs and other aspects of care. It was also reported as effective in communicating patient care status to other staff. However, completing the checklist was also described as a task which could be overlooked during busy periods. During implementation, the checklist was promoted to staff in ways that obscured its core function of maintaining patient safety.

**Conclusions:**

The Emergency Department Safety Checklist can support staff in maintaining patient safety. However, it was not fully recognised by staff as a core component of everyday clinical practice.

**Relevance to clinical practice:**

The Emergency Department Safety Checklist is a response to an overcrowded environment. To realise the potential of the checklist, emergency departments should take the following steps during implementation: (a) focus on the core function of clinical safety, (b) fully integrate the checklist into the existing workflow and (c) employ a departmental team‐based approach to implementation and training.

## 1 INTRODUCTION

The UK, and other countries, have seen an unprecedented increase in demand for Emergency Department (ED) services in recent years (Di Somma et al., 2015; Maguire, Dunn, & McKenna, 2016). This has led to crowding where the number of patients exceeds the capacity for which an ED is designed and resourced (Royal College of Emergency Medicine, 2015). As demand and workload rise, staff can become overwhelmed by tasks (Boreham, Shea, & Mackway‐Jones, 2000), and the risk of undetected patient deterioration increases (Ramlakhan, Qayyum, Burke, & Brown, 2015). Patients sometimes have to wait several hours in the ED for diagnostic tests and inpatient beds. The number of healthcare staff who interact with a patient increases and so does the risk of communication errors (Maguire et al., 2016; Ong, Biomede, & Coiera, 2011). These factors jeopardise the ED's capacity to deliver safe and high‐quality care (Kallberg, Ehrenberg, Florin, Ostergren, & Goransson, 2017).

To address these patient safety issues, an ED safety checklist (see Appendix S2) was developed by University Hospitals Bristol NHS Foundation Trust in 2014 (The Kings Fund, 2018; Redfern et al., 2018). In association with the West of England Academic Health Science Network (WEAHSN), the checklist was adopted by six hospitals in the region and has subsequently been recommended for use across NHS EDs in England (NHS Improvement, 2018). The checklist is designed to ensure patients' clinical condition is regularly monitored, deterioration is identified quickly, and care needs are met.

This paper explores (a) staff views, perceptions and use of the ED Safety Checklist in two UK hospital EDs and (b) the implementation strategies employed in the two sites.

## 2 BACKGROUND

The ED Safety Checklist is a time‐based framework for nursing and care tasks completed for every patient, except those with minor complaints. It is a paper‐based document which enables staff to record that vital signs have been measured and acted upon appropriately (Johnson, Mueller, & Winkelman, 2017); early warning scores have been calculated; and other clinical investigations such as electrocardiograms, X‐rays and blood tests have been carried out (see Appendix S2 for an example of the ED Safety Checklist). The checklist also documents other aspects of care such as offering drinks/food, contacting relatives and assessing pressure areas. Most vital sign measurements on the checklist are required hourly or more frequently if clinically indicated.

These aspects of care are established elements of clinical practice, but during times of crowding in the ED staff find it difficult to meet competing demands (Royal College of Emergency Medicine, 2015). The purpose of the ED Safety Checklist is to ensure patients' clinical condition and care are regularly monitored and deterioration is identified quickly. At the same time, it provides a quick and accessible summary of the status of patient care tasks to any member of the ED staff. It is also intended to support temporary staff who are increasingly required to work in EDs as the established ED workforce numbers are no longer sufficient to meet the demand (Evans & Ward, 2017).

The ED Safety Checklist was developed initially as a quality improvement project (Redfern et al., 2018) and subsequently rolled out across six hospitals in the West of England. Small local adaptations were made at each hospital to reflect particular ED workflows and processes. The roll‐out was led by the WEAHSN who brought together clinical and nonclinical representatives from seven regional hospital EDs to work collaboratively, sharing ideas, experiences and data around implementation (WEAHSN, 2018).

The adoption and use of checklists in health care and other safety–critical environments are dependent on implementation strategies that engage with a range of professional, organisational and cultural factors (Dixon‐Woods, Bosk, Aveling, Goeschel, & Pronovost, 2011; Dixon‐Woods, Leslie, Tarrant, & Bion, 2013). Our study of the ED Safety Checklist built on this literature and explored the factors that impacted on the use and take‐up. We employed observation and interviews in a focused ethnography (Higginbottom, Pillay, & Boadu, 2013) to (a) identify the strategies used to incorporate the checklist into clinical practice and (b) assess whether the ED Safety Checklist was recognised by ED staff as an effective framework to support safe care.

## 3 METHODS

The research took place between June and September 2017 in the EDs of two UK National Health Service (NHS) hospitals that had adopted the ED safety checklist in December 2015. The EDs were selected to represent different EDs and allow comparison between them, one was a teaching hospital and the other nonteaching. We employed two data collection strategies: nonparticipant observation (hereafter referred to as observation) of the use of the ED Safety Checklist in real‐time, and semi‐structured interviews with staff who used the checklist or had a role in its implementation. The observations enabled an understanding of the way the checklist was used in practice which in turn informed the subsequent staff interviews.

Contact with the EDs was initiated with lead clinicians who informed staff via email and internal staff meetings about the purpose of our study and how it would be carried out. Orientation visits were undertaken at each ED by researchers to understand the layout of the departments and to be introduced to key members of staff. Researchers' roles were described as independent and focused on the use of the ED checklist rather than on auditing or assessing professional performance. Prior to a period of observation, researchers explained the study to staff on a one‐to‐one basis and gained verbal consent to observe them using the ED Safety Checklist. Patients were informed about the presence of researchers across the ED where patients were admitted, triaged and treated.

The observation sessions covered a range of days of the week and times of day and night to enable us to see the ED Safety Checklist in use during different levels of demand. Researchers were mindful of patient privacy and of avoiding interference with clinical work. Observations were undertaken in 3‐hr episodes at various time points over two consecutive weeks to a total of 33 hr at each ED (see Table 1).

**Table 1:** Participants and data collection

Interviewees by role	ED1 Participants (*n*)	ED2 Participants (*n*)	Total
Nursing Assistant	3	6	9
Nurse	2	4	6
Agency Nurse	2	0	2
Senior Nurse	4	0	4
Senior ED Staff	2	3	5
*Range minutes*	*6–39*	*10–34*	*6–39*
Total	13	13	26

Observations	ED1 (hr)	ED2 (hr)	Total
Morning (08:00–12:00)	16	10	26
Afternoon (12:00–18:00)	10	16	26
Evening/Night (18:00–02:00)	7	7	14
Total	33	33	66

Interviewee role categories	
Nursing Assistant	Healthcare assistant, assistant practitioner and nonclinical support staff
Nurse	Staff nurses and senior staff nurses
Senior Nurse	Nurse practitioners and sisters
Senior ED staff	Consultants, matrons and specialist nonclinical staff involved in implementation

Staff were purposefully sampled for the interviews to capture a range of experiences of working with and implementing the ED checklist. Sampling criteria were nursing staff who were required to use the ED checklist from auxiliary/assistant level to registered and senior nurses; clinical and nonclinical staff involved in the implementation of the checklist; and doctors or consultant leads in the ED. Interviews took place in private rooms, and staff gave written informed consent. We used a topic guide (see Appendix S3) which was developed from our research questions and insights from our observation data. Thirteen interviews were conducted at each ED with a range of staff and lasted between 6–39 min (see Table 1). On two occasions, staff agreed to be interviewed but were too busy to participate at the scheduled time and were unable to reschedule.

Observation and interview data were collected at each ED by JB (male), TS, HB and JK (female) who are all social scientists (PhDs) with a minimum of 6‐year experience of conducting qualitative research on a range of health topics.

Data were reviewed throughout the data collection period at research team briefings until we were satisfied that we had reached data saturation (Sandelowski, 1995) such that our observations had captured a full range of actions associated with the checklist and interviews ceased to reveal new data.

Handwritten observation notes were typed up, and interview audio files were transcribed verbatim by an accredited transcription company. All data were fully anonymised and imported into NVivo 10 (QSR International) and analysed thematically (Braun & Clarke, 2006). We used a data‐driven inductive approach to identify patterns and themes across the data set and by research site. Analysis commenced with open coding. JB and TS each coded a sample of early transcripts and field notes and jointly developed an initial coding framework. Following a further round of double coding, the coding framework was agreed and applied to the full data set. The analysis proceeded by developing broader categories through comparison across the transcripts and field notes and identifying higher‐level recurring themes. Members of the study team met regularly to discuss analysis.

The research was approved by a United Kingdom University health research ethics committee and the Health Research Authority in England. It adhered to the COREQ guidelines for qualitative research (see Appendix S1).

## 4 RESULTS

Findings are reported under two main themes: (a) the ED Safety Checklist: documenting care in the ED and (b) implementing and embedding the ED Safety Checklist. Data excerpts in the result section are tagged by role (as described in Table 1); study ED; study participant ID for interviewees or “field notes” where they refer to excerpts from researchers' notes.

### 4.1 Part 1: The ED Safety Checklist—documenting care in the ED

#### 4.1.1 The ED Safety Checklist and patient safety

The ED Safety Checklist was completed by nurses and nursing assistants. Many described it as a supportive framework to prompt or remind them to undertake and document clinical observations and care tasks. This information was collated on the ED checklist, providing real‐time updates on completed and outstanding tasks. This enabled staff to track patients' clinical condition, along with their comfort and care needs, and thereby maintain patient safety in a time‐pressured environment:

It's actually quite good because you monitor a patient every hour and you know what's happening with your patient. So it's very unlikely the patient will be neglected or forgotten about. (Nurse, ED1: ID10)

I think it is important, it does keep them safe, especially if it's filled out correctly and you know their pressure areas have been checked and they've had their ECG and you know that everything's been done because it's on here, it is better. (Nursing Assistant, ED2: ID24)

One of the aims of the ED Safety Checklist was to provide a framework for managing tasks for staff unfamiliar with the ED environment (e.g. new or temporary staff) and enable them to work safely without requiring constant supervision:

Being new here and coming into this environment I didn't really have a clue what I was doing! So, I actually found this very helpful because it literally told me exactly what I needed to do like they needed to have the triage, their wristbands, get changed, make sure that pressure areas and all that are sorted, have their ECG done, otherwise I'd probably be a bit of a mess. (Nursing Assistant, ED2: ID21)

#### 4.1.2 The ED safety checklist—a communication tool?

Increasing numbers of people attending EDs mean that patients tend to stay longer in the ED and are more likely to remain across shift changeovers for nursing staff and doctors. The ED Safety Checklist was designed to improve the handover of care in these circumstances by quickly communicating the clinical and care status of the patient. Staff found the Checklist to be effective in this respect, particularly when face‐to‐face contact was not possible:

The next shift when they come on, or if you've gone on your break and someone's taken over, they can just quickly look at it [ED Safety Checklist] and think “Right, that's all done, all I've got to do is an ECG or that's the only thing that's missing” … You haven't got to keep communicating the same thing to a hundred people. (Nursing Assistant, ED2: ID16)

#### 4.1.3 The ED Safety Checklist and nonclinical care

The ED Safety Checklist incorporated aspects of nonclinical care such as offering refreshments and contacting relatives. Nursing Assistants in ED 1 and nonclinical support staff in ED 2 routinely used the ED checklist to record that patients had been offered food and drink and whether it had been accepted. The checklist formalised these aspects of care making them less vulnerable to being overlooked.

Things like [contacting] next of kin or refreshments, although they're not clinical, they are really important and it's really nice that there is a prompt there. Because it's surprising how long patients go and think ‘oh, no one knows we're here' especially the elderly. (Senior Nurse, ED1: ID12)

#### 4.1.4 The ED Safety Checklist: completion versus workload

Some staff reported that the ED Safety Checklist added an additional task of documentation that was perceived as duplication; clinical observations were recorded as usual on an “observations chart” and then ticked off as complete on the checklist. This represented an additional task in a busy environment:

It's just another box to tick, just more work, I do it all on there [points to the clinical observation chart] and then I do it all down here [points to the ED Safety Checklist]. (Nurse, ED1: field notes)

The biggest reported challenge in terms of completing the checklist was the demand on staff time. When the ED was busy, staff consistently described it as hard to complete within the specified time schedule. It was notable that staff prioritised completion of the clinical observations chart while documenting those actions on the ED Safety Checklist was seen as the expendable element when busy:

I would lose sleep over missing writing down some obs whereas I wouldn't if the obs were written down but I didn't do the checklist. (Nursing Assistant, ED2: ID23)

The use of the checklist goes out of the window when the department is busy. (Nursing Assistant, ED2: field notes)

### 4.2 Part 2: Implementing and embedding the ED Safety Checklist

We have described a degree of ambivalence about the use of the ED safety checklist and the perceived impact on workload. In the section below, we explore the relationship between the implementation strategies used and the impact on the perception and take‐up of the checklist.

#### 4.2.1 How the ED Safety Checklist was promoted

While both EDs highlighted the patient safety aspects of the checklist, the implementation was accompanied by other key narratives. A recurring theme was that of the ED Safety Checklist as a tool to provide evidence that care had been given in the event of a complaint:

It's to cover yourself more than anything … this is a way of people looking back and seeing it and it's a prompter and a reminder for yourself as well if you ever had a case that had to go to court or something. (Nurse, ED2: ID25)

Staff captured this perspective in the phrase, “if it isn't written down, it didn't happen.” This phrase was used at both EDs but as a driver it was particularly evident in ED1 where it was a feature of the implementation strategy, it became a key message to encourage uptake and use:

I guess it's kind of scaring them a little bit but it's a reality that if you're looking after a patient and something happens and you haven't documented everything, or you haven't looked after them appropriately for whatever reason, it might be that you couldn't because there's only one of you, then you might have to go to court and you have to cover your back. (Senior Nurse, ED1: ID08)

In contrast, at ED2 the implementation message focused on the ED Safety Checklist as a vehicle to provide evidence to senior hospital managers and external inspectors for the high standard of care being delivered:

That [ED checklist] document is our documentation of what care we've given them. And so, if that hasn't been filled out then it doesn't look great for the ward either, and it doesn't look great for us, because it looks like we haven't given any care. (Nurse, ED2: ID19)

If you're too busy to fill out the checklist then that's a bigger problem ‘cause the fact is if you're not too busy to take obs you're not too busy to run a checklist' but it's a case of whether it's seen as a priority or not. And part of that is how we've marketed it, it's seen as an administrative document rather than necessarily a patient safety document so they will do the obs and they will fill that out but in terms of them coming back over to write that they've done it, because that's seen as an administrative task. (Senior ED, ED2: ID20)

Both EDs emphasised the value of documentation. At ED1, there was more emphasis on the defensive medico‐legal framework provided by the checklist, and in ED2, the emphasis was on the ED Safety Checklist as a tool for providing evidence of care.

#### 4.2.2 Integrating the ED Safety Checklist—presentation

There were differences between the two EDs in how the checklist was adapted and formatted. In ED2, optimising presentation was given greater support from the hospital leadership and infrastructure, particularly in relation to formatting and printing the document to align with existing paperwork:

We spent a bit of time making it very beautiful …we wanted to launch it all in a booklet that was colourful that was user friendly and that would be quite attractive to complete. (Senior ED Staff, ED2: ID15)

We did also get support from the Quality Improvement Team as well … they've supported us and given us money … to get it all done nicely. (Senior ED Staff ED2: ID20)

In ED1, there was less support for formatting and integration. At the time of the research, the checklist was separate from the clinical observation chart and stapled to existing paperwork:

Initially it wasn't attached [in ED1] … quite quickly we came up with ‘right let's staple it to the observation chart'‐ it's there then, you know then at least people might use it and not have to struggle. (Senior Nurse, ED1: ID08)

#### 4.2.3 Integrating the ED Safety Checklist—training

Staff described different approaches to training. At ED1, it was the responsibility of a senior nurse, who provided individual or small group training whenever possible. Staff valued the individual contact, but the ED Safety Checklist became associated with one individual who felt under pressure to fulfil the implementation of the checklist with little available support from senior staff:

I had a little presentation so it was just sort of ad‐hoc where we were not too busy just grabbing a couple or one and just doing a bit of a talk through why we were doing it, where it had come from, how to fill it in that kind of thing….I was doing it in the department. Occasionally you can find a spare computer, it was literally just when I could, when it wasn't too busy. (Senior Nurse, ED1: ID7)

In contrast, at ED2, implementation was described as a team operation and there was a department‐wide approach to training. The hospital education teams were utilised along with ED Safety Checklist training sessions at formal away days for key nursing staff. Senior medical staff in the ED actively promoted use of the checklist, reinforcing it as a collectively owned departmental initiative.

It embedded pretty quickly but [we] did a lot of education real‐time, we all did; we used it as safety briefing, we launched it at band 7 and band 6 away days and then we used our education team to put it out there, we let people play with it and then we started to maintain what our expectations really [were] about its completeness. (Senior ED Staff, ED2: ID15)

## 5 DISCUSSION

Our study provides insight into the early implementation of a patient safety checklist that has been designed in response to a profound shift in the demand on EDs in the UK (Redfern et al., 2018) especially in the winter periods where crowding undermines patient safety (Maguire et al., 2016). There is currently no published research‐based evidence that the ED Safety Checklist improves patient safety, but it is premised on the basis of a substantial body of evidence which demonstrates the value of checklists in health care and in industries where safety is paramount (Catchpole, 2015; Hales, Terblanche, Fowler, & Sibbald, 2008; Kapur, Parand, Soukup, Reader, & Sevdalis, 2016; Kohn, Corrigan, & Donaldson, 2000). Research has also shown clinicians' relationships with and uptake of checklists depends on a variety of factors: beliefs and expectations about benefits, previous experiences, checklist complexity, involvement in the checklist development and the orientation of clinical teams (van Daalen, Geerlings, Prins, & Hulscher, 2016; Russ et al., 2015; Singer et al., 2016).

Our study follows on from the quality improvement report undertaken by Redfern et al. (2018) which documents the initial development of the ED Safety Checklist at one hospital. Our results show that staff recognised the potential of the checklist for improving patient safety. It was designed so that staff could maintain regular contact with their patients regardless of crowding pressures. In many respects, our data indicated that the checklist had been successful in this. Staff felt that it worked well to prompt and remind them of clinical and nonclinical care tasks and that it served as an effective means of communication when care was transferred between different staff members.

However, staff also expressed frustration that the checklist duplicated existing clinical documentation which, as Aveling, McCulloch, and Dixon‐Woods (2013) note, increases the risk that a checklist is perceived as a “bureaucratic intrusion.” A critical limitation that undermined the core objective of the checklist was that staff reported that it was most likely not to be used when demand was high, when patients were more vulnerable to undetected deterioration. There is a tension around the value attributed to the ED Safety Checklist by staff. On the one hand, it was recognised as useful, and on the other hand, it was seen as expendable, something nonessential in high demand situations. This resonates with Kocman, Stöckelová, Pearse, and Martin (2019) who outline the different logics, or purposes, of a checklist (check, prompt, audit and record) and the tensions that emerge between them which can undermine implementation and use. We explore potential causes of ambivalence and tension in the use of the ED safety checklist below.

Research has highlighted the importance of staff being fully aware of and having a shared understanding of the purpose of checklists (Clay‐Williams & Colligan, 2015; Hales et al., 2008; Kallberg et al., 2017). Initiatives such as “intentional rounding,” which bears many similarities with the ED Safety Checklist, are based on clinical staff delivering care within specified time frames in a proactive manner. Rounding studies highlight the importance to successful implementation of developing a widely shared understanding of safety tools along with full engagement by all staff (Christiansen et al., 2018; Forde‐Johnston, 2014; Mitchell, Lavenberg, Trotta, & Umscheid, 2014; National Nursing Research Unit, 2012; Toole, Meluskey, & Hall, 2016). It is notable that staff in our study did develop shared understandings and meanings, but these differed from the primary purpose of the ED Safety Checklist which was to promote patient safety via structured monitoring.

We argue that implementation strategies associated with the ED Safety Checklist may have contributed to undermining its core clinical purpose. In both EDs, implementation was accompanied by an administrative discourse: as a protective medico‐legal document in ED 1 and as a means of promoting and protecting departmental status in ED2. These divergent perspectives reflect a different emphasis given to the purpose of the ED safety checklist during the implementation phase and illustrate how different meanings can be associated with the same checklist. These approaches to promoting the checklist to staff were employed strategically to encourage take‐up. However, the emphasis on administrative discourses undermine the clinical status of checklists and make them less likely to be completed during busy times (Aveling et al., 2013; Hallam et al., 2018).

We have described differences between the EDs associated with the way that the checklist was promoted to staff and these both had the same effect—to undermine the core patient safety message. However, we also observed differences between the two EDs which had a substantive impact on the integration of the checklist into the hospital workflow. ED2 made extensive efforts to integrate the form with existing paperwork and workflow in an effort to avoid the checklist being seen as “just another form.” They were able to mobilise support from the hospital leadership and infrastructure to embed the checklist in existing paperwork. In contrast, in ED1 it was attached as a separate piece of paper and was vulnerable to being disassociated from the core clinical documentation. The differences between the EDs were also evident around training, a significant factor in checklist implementation (Puttick, Speirs, Gibson, Tadjkarimi, & Ahmad, 2016), in that ED2 were able to draw on training resources supported by the hospital infrastructure that were not available in ED1. More than this, in ED2 the implementation was mobilised around a team that included senior nursing staff and consultants whereas in ED1 it was led by a senior nurse. The difference led to the checklist being perceived as a departmental project in ED2 which gave implementation a greater momentum (Table 2).

**Table 2:** Summary results and implications

Implementation action	Implementation benefit
Promoting the ED Safety Checklist—focus on the primary function of the checklist, “to avoid undetected patient deterioration”	The checklist becomes associated with core clinical work and increases the likelihood of full completion during busy periods
Mobilise support from across the ED including doctors and senior staff	The checklist is more likely to be identified as integral to departmental workflow
Secure support from hospital infrastructure and resources for formatting, presentation and integration of the ED Safety Checklist	Improved integration with existing documentation reduces the perception of it being, “just another form”
Dedicated training time for staff and implementation team	Greater scope for communicating the purpose of the ED safety checklist, how it can support patient safety
Implementation should be team‐based	Strengthens the association between the ED Safety Checklist and the Department which gives a greater level of legitimacy to the checklist

It is not possible to make generalisable claims from our data about the impact on implementation linked to hospital type: teaching versus nonteaching. However, we can see that access to resources around training and integration with existing documentation enabled ED2 to go further in integrating the checklist into existing workflows. The way that ED2 was also able to mobilise the ED Safety Checklist as a collective departmental project, rather than one identified with an individual, gave implementation a greater momentum.

## 6 LIMITATIONS

To fully evaluate the impact of the ED Safety Checklist on patient safety, a quantitative study measuring clinical outcomes will be necessary. We also recognise that our data did not enable evaluation of the impact on patient experience.

As a small‐scale qualitative study, there are limitations about the transferability of the findings. However, crowding and patient safety are issues faced across the UK, and internationally. We suggest that our results, particularly around implementation, are directly relevant to Emergency Departments experiencing crowding and the NHS England recommended introduction of a patient safety checklist in the UK (NHS Improvement, 2018). They also resonate with the research literature around changing practice in safety‐critical environments including healthcare settings.

It is possible that an “observer effect” (McDonald, 2005) led to staff changing behaviour during periods of our observation. We mitigated this by emphasising that we were not auditing or examining their practice.

There was a risk that research staff could inaccurately interpret and record ED activity during observations due to unfamiliarity with the setting. We minimised this by using 3 researchers in each ED who debriefed and checked in with other researchers after each session and by iteratively building questions about what had been observed into interviews.

## 7 CONCLUSION

Our research has highlighted tension in the use of the ED Safety Checklist. On the one hand, the checklist has been identified by staff as a useful framework for structuring clinical activity in a safe and consistent way. On the other hand, staff reported a tendency to not use the checklist when the ED was deemed to be too busy. For the checklist to fulfil the function of maintaining patient safety, it should be used during busy periods when patient safety is at higher risk. Therefore, implementation strategies should focus on the core patient safety function of the checklist and integration into existing documentation and workflow.

## 8 RELEVANCE TO CLINICAL PRACTICE

The ED Safety Checklist has the potential to support healthcare staff in EDs to monitor patients regularly and so better maintain patient safety. Its adoption across the UK has been recommended by NHS England (NHS Improvement, 2018).

When implementing the ED Safety Checklist, we recommend that EDs should

- focus on the core function of the checklist to improve patient safety rather than discourses with an administrative or bureaucratic association which can undermine its use,
- make available hospital resources such as design and printing that can support the development and integration of the checklist into the local workflow and improve its recognition and
- mobilise a team‐based approach to implementation and training that draws on a wide range of staff including consultants and senior nurses so that the checklist is recognised as having departmental backing.

## CONFLICT OF INTEREST

Emma Redfern, one of the authors, is a Consultant in Emergency Medicine and contributed to the original design of the ED checklist. Ann Remmers and Emma Redfern are members of the West of England Academic Health Science Network who supported the roll‐out and implementation of the ED Checklist in their region. Neither Emma Redfern or Ann Remmers had access to nonanonymised data nor did they participate in the data analysis stage, and they did contribute to the background, discussion and conclusions of the paper.

## AUTHOR CONTRIBUTIONS

Emma Redfern, Sabi Redwood, Jon Banks and Ann Remmers were involved in the design of the study. Jon Banks, Tracey Stone, Joanna Kesten and Heather Brant collected the data for the study. Jon Banks and Heather Brant undertook the background literature search. Tracey Stone and Jon Banks undertook the data analysis and drafted initial versions of the paper. Tracey Stone, Jon Banks, Emma Redfern, Ann Remmers, Joanna Kesten, Heather Brant and Sabi Redwood all made substantive contributions to the final submitted paper including the background, methods, results, discussion and conclusion.
