# Supplementary material for: The introduction of a safety checklist in two UK hospital emergency departments: A qualitative study of implementation and staff use
Source: J Clin Nurs. 2020 Jan 30;29(7-8):1267–75. doi: 10.1111/jocn.15184 (PMC7161913; doi:10.1111/jocn.15184)
Supplement: Supplementary file 1 [file JOCN-29-1267-s001.pdf]

Emergency Department Safety Checklist

Patient Label here...

Date____________________ **Time Booked in**_____________

	Action	Time	Initials	Comments
**1st hour completion time : :**	Assessment/Triage			
	Vital signs measured + NEWS recorded			
	ECG recorded (within 10 minutes)			
	*ECG reviewed by Dr (within 30 minutes - **time** on ECG)*			
	Undressed and gown			
	Wristband			
	Pain score assessed			
	Analgesia administered (if appropriate)			
	Infection control screening			
	Sepsis suspected (Temp < 36° or > 38°C, HR > 90 or RR > 20)			
	IV access + care plan			
	Blood tests			
	Imaging (Stroke, # NOF within 1 hour)			
	Specific Pathway Triggered (see box 1)			
	PFC informs CST - specialty bed required			
	Pathway commenced (e.g. Stroke, DKA, NOF, GI bleed, Sepsis)			
**2nd hour completion time : :**	Vital signs measured + NEWS recorded			
	Pain score assessed			
	Analgesia administered (if necessary)			
	Next of kin aware			
	Patient has dementia (*This is me* commenced)			
	Refreshments offered (if not NBM)			
	**Pressure Area Care:**			
	Assessment undertaken			
	Care plan commenced (as appropriate)			
	**Patient good to go:**			
	Patient ready for transfer			
	Specialty bed confirmed			
**3rd hour completion time : :**	Vital signs measured + NEWS recorded			
	Pain score assessed			
	Analgesia administered (if necessary)			
	Refreshments offered (if not NBM)			
	Review by senior doctor			
	Regular medication administered (if appropriate)			
**4th hour completion time : :**	Vital signs measured + NEWS Recorded			
	Pain score assessed			
	Analgesia administered (if necessary)			
	Refreshments offered (if not NBM)			
	Regular medication administered (if appropriate)			
**Referrals & Pathway/Speciality Triggers if required**	Adult safeguarding referral			**Box 1 - Specialty Bed Trigger:**
	Child cause for concern referral			
	Mental health matrix completed			Stroke/TIA ☐ Stroke Unit (B504)
	Mental Health referral			Upper GI Bleed ☐ Ward 11 (B404) or MAU (A300)
	Domestic or sexual violence Yes / No			DKA ☐ MAU (A300) or ITU/HDU
	IDSVA referral			NIV ☐ Respiratory (A522) or MAU (A300)
	Paddington Alcohol Test Yes / No			Chest Drain ☐ MAU (A300), Respiratory (A522) or BHI/700
	Referral to Alcohol Clinical Nurse Specialist			# NOF ☐ T&O (A609)
	Referral to Drug Clinical Nurse Specialist			Tracheostomy ☐ Ward 700, A522 or ITU/HDU/CICU

Authors: Jason Lugg & Hayley Thomas (November 2014)
