# Supplementary material for: The introduction of a safety checklist in two UK hospital emergency departments: A qualitative study of implementation and staff use
Source: J Clin Nurs. 2020 Jan 30;29(7-8):1267–75. doi: 10.1111/jocn.15184 (PMC7161913; doi:10.1111/jocn.15184)
Supplement: Supplementary file 2 [file JOCN-29-1267-s002.docx]

**Appendix S2**

### **Topic guide – ED Checklist staff**

**Interview purpose** – To understand how the implementation of the ED Checklist has been from their point of view and their thoughts on how it’s working now.

**Consent** – researcher to explain the purpose of interview; provide opportunity to ask questions; explain interview will be audio recorded; anonymous quotations may be used in future reports and publications without sources being identifiable;

Turn on recorder: verbal consent from participant

## Part 1 – your role

How long have you been working in ED? 🡺 How long in this Department?

Could you describe your role in this ED and how you use the ED Checklist?

## Part 2 – introducing ED Checklist

Describe roll-out of ED Checklist in this Department 🡺 Date introduced?

Were you involved in the introduction or role out of the ED checklist?

Initial reaction of staff in differing roles 🡺 factors that encouraged/discouraged its use?

How and when is the ED checklist administered?

Tell us about any initiatives that have been used to get the ED checklist adopted – were these linked to any national or local organisations/initiatives?

What was the role of the WEAHSN in getting the checklist adopted? Is there/ has there been any training provided on using the ED Checklist?

What are the benefits of using the ED checklist?

What are the problems with using the ED checklist?

What has been the impact on patient safety since using the ED checklist? Can you give us an example?

Do you give feedback to patients about the ED checklist? What are patients’ reactions to this?

What improvements could be made to the ED checklist (either checklist content or method of administration)?

Anything to add about the ED checklist that we haven’t covered?
